# Expression of free fatty acid receptors in the liver of periparturient dairy cows supplemented with essential fatty acids and conjugated linoleic acid

**DOI:** 10.3168/jdsc.2025-0867

**Published:** 2025-12-13

**Authors:** Tainara C. Michelotti, Alyssa Imbert, Arash Veshkini, Guillaume Durand, Harald M. Hammon, Muriel Bonnet

**Affiliations:** 1 INRAE, Université Clermont Auvergne, VetAgro Sup, UMR Herbivores, 63122 Saint Genès Champanelle, France; 2 Research Institute for Farm Animal Biology (FBN), 18196 Dummerstorf, Germany; 3 Feed and Food Department, Bordeaux Sciences Agro, 33170 Gradignan, France

## Abstract

Free fatty acid receptors (FFAR) are molecular sensors involved in the regulation of energy metabolism. Free fatty acid receptors are expressed in the bovine liver, although their biological functions are not fully understood. Our objectives were to study the expression of hepatic FFAR in periparturient dairy cows supplemented or not with a mixture of essential fatty acids (EFA) and CLA, and to investigate potential associations between FFAR and metabolic adaptation during the transition period. Multiparous Holstein cows received abomasal infusions of either coconut oil (control; n = 8) or a mixture of EFA and CLA (EFACLA; n = 8) from −9 to 9 wk relative to parturition. Liver samples were collected at −3, 0, 4, and 9 wk relative to parturition. We quantified the liver expression of FFAR (*FFAR1*–*4* and *GPR84*) and peroxisome proliferator-activated receptor delta (*PPARD*) by real-time quantitative PCR. Repeated-measurement correlations and multilevel multiple factor analysis (MFA) were used to investigate the links between FFAR and other metabolic parameters (i.e., energy balance, blood metabolic indicators, liver proteomics, and liver gene expression). All targeted FFAR were expressed in the liver, except for *FFAR4*. We found no effects of EFACLA or interactions with time for the expressed FFAR. *FFAR1*, *FFAR2*, and *GPR84* expression decreased from −3 to 9 wk relative to parturition, whereas *FFAR3* remained constant from −3 to 4 wk, then decreased at 9 wk postpartum. We observed strong correlations between FFAR, and moderate correlations between FFAR and *PPARD.* Multivariate (MFA) and univariate (correlation) analyses revealed weak links between FFAR liver expression and other metabolic parameters (e.g., *IGFBP3* liver expression and plasma IGFBP-2). Downregulation of FFAR in the liver from pre- to postpartum may prevent receptors hyperactivation during periods of high free fatty acid concentrations. Physiological relevance and individual contributions of FFAR to the hepatic metabolism require further investigation.

## Highlights

• Liver *FFAR1*, *FFAR2*, and *FFAR3*, and *GPR84* were downregulated from pre- to postpartum.
• Supplementation with EFA and CLA did not affect FFAR.
• Expression of liver FFAR were strongly correlated.
• *PPARD* expression peaked at calving and was moderately correlated with FFAR.

## Graphical Abstract

**Figure:**
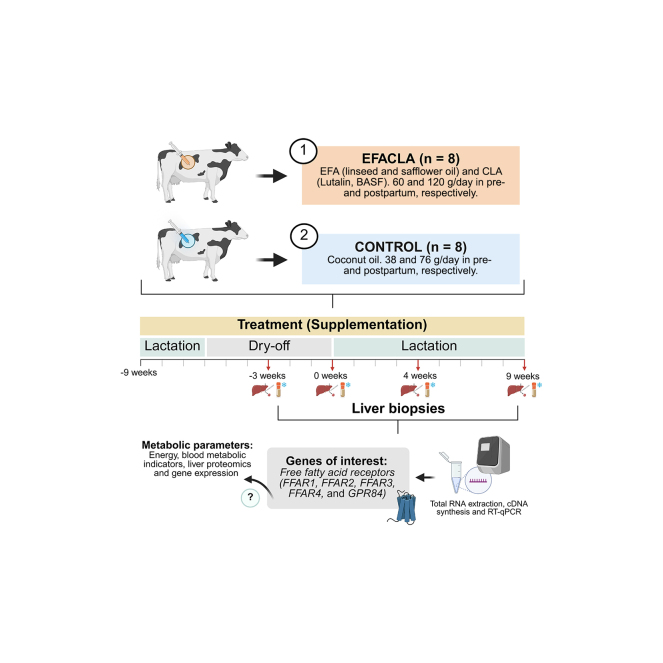
**Summary:** We studied the expression patterns of hepatic free fatty acid receptors (FFAR) in dairy cows around parturition and early lactation, and the effects of supplementation with essential fatty acids (EFA) and conjugated linoleic acid (CLA) on their expression. Additionally, we investigated the association between hepatic FFAR and other metabolic parameters measured during the transition period. All targeted FFAR were expressed in the liver, except for *FFAR4*. We found no effects of EFACLA or interactions with time for the expressed FFAR. Expression of *FFAR1*, *FFAR2*, and *GPR84* decreased from -3 to 9 weeks relative to parturition. *FFAR3* levels remained constant from -3 to 4 weeks, then decreased at 9 weeks postpartum. We found strong correlations between FFAR and moderate correlations between FFAR and *PPARD*. We observed weak links between liver FFAR and other metabolic parameters. Free fatty acid receptor downregulation from pre- to postpartum may prevent receptor hyperactivation during periods of high free fatty acid concentrations. cDNA = complementary deoxyribonucleic acid; RT-qPCR = real-time quantitative polymerase chain reaction. Figure created in BioRender.com.

The liver plays key roles in metabolic and endocrine adaptations that occur during the transition from late pregnancy to early lactation. For instance, it contributes to the systemic somatotropic axis regulation, oxidation of adipose-mobilized fatty acids (**FA**), and gluconeogenesis (Chilliard, 1999). In addition, the liver expresses free fatty acid receptors (**FFAR**), molecular sensors of free fatty acids (**FFA**) with demonstrated roles in the regulation of energy metabolism in monogastric animals (Hara et al., 2013). Free fatty acid receptors, including FFAR1–4 and GPR84, are members of the G protein-coupled receptors (Briscoe et al., 2003; Brown et al., 2003), a family of receptors involved in signaling across cellular membranes.

In monogastrics, FFAR have been studied as potential therapeutic targets for various health disorders, including liver disease and diabetes (Kimura et al., 2020; Secor et al., 2021). For instance, FFAR2 and FFAR3, receptors of short-chain FFA, regulate hepatic lipid metabolism in mice (Shimizu et al., 2019; Aoki et al., 2021), whereas the activation of FFAR1, a receptor of medium and long-chain FFA, improves hepatic insulin sensitivity and β-oxidation through the activation of the peroxisome proliferator-activated receptor delta (PPARD) in human HepG2 cells (Wu et al., 2012).

In bovines, however, the biological function and metabolic impact of hepatic FFAR remain poorly understood. A recent study suggested an association of FFAR1 with imbalances in liver metabolism in transition dairy cows, through unknown mechanisms (Aguinaga Casañas et al., 2017). Agrawal et al. (2017) proposed that FFAR expression provides an additional level of control over receptor activation, underscoring the importance of studying FFAR transcription. The current literature indicates that *FFAR1* is downregulated in the liver following parturition, whereas *GPR84* and *FFAR2* levels remain unchanged (Friedrichs et al., 2014; Agrawal et al., 2017). Overall, the concomitant expression of the different FFAR (*FFAR1–4*, and *GPR84*) has not yet been investigated within the few studies examining FFAR liver expression around parturition.

Essential fatty acids (**EFA**; α-linolenic and linoleic acids) and CLA are known agonists of both FFAR1 and 4. Essential fatty acids are among the most potent FA agonists for the human and mouse receptors (Ulven and Christiansen, 2015) and linoleic acid directly activates FFAR1 in bovine neutrophils (Manosalva et al., 2015). In dairy cows, EFA and CLA were supplemented as a strategy to improve immune function and energy status during the transition period (Gnott et al., 2020; Vogel et al., 2020). It is not yet known how the expression of FFAR in the liver is modulated by EFA and CLA supplementation and how FFAR may contribute to the metabolic adaptations around the time of parturition.

Our current study is part of a larger research project investigating the effects of EFA and CLA abomasal infusions on dairy cows (Gnott et al., 2020; Vogel et al., 2020, 2021; Veshkini et al., 2022). These studies have demonstrated that compared with the control cows, cows supplemented with CLA and EFA had a better energy balance (**EB**; −7.6 vs. −23.0 ± 32.4 MJ NEL/d [mean ± SD]) due to lower lipid secretion in milk (2.44% vs. 4.36% ± 1.46%) and lower adipose tissue (**AT**) mobilization, as indicated by the levels of nonesterified fatty acids (**NEFA**) in the blood (311.6 vs. 488.8 ± 376.2 µmol/L). Furthermore, EFACLA altered the liver proteome with main effects on the activation of cytochrome P450 pathways, which are related to liver FA ω-oxidation, among other functions.

The objective of our study was to determine the expression patterns of hepatic FFAR (*FFAR1–4* and *GPR84*), as well as *PPARD* as part of their downstream regulation, around parturition and early lactation, and to investigate the effects of supplementation with EFA and CLA on their expression. We also aimed to investigate the potential roles of hepatic FFAR in the metabolic adaptation of dairy cows during transition. To this end, we examined time effects on FFAR expression and associations between FFAR expression and other metabolic parameters (i.e., EB, blood metabolic indicators, liver proteomics, and liver gene expression), determined in the present study or as described by the companion articles (Gnott et al., 2020; Vogel et al., 2020, 2021; Veshkini et al., 2022).

Animal trial was carried out as described previously (Vogel et al., 2020). Briefly, 16 multiparous Holstein cows were abomasally infused with either coconut oil (control, n = 8; Bio-Kokosöl, catalog no. 665, Kräuterhaus Sanct Bernhard) or EFA+CLA, a combination of linseed oil (DERBY Leinöl Cat. No. 4026921003087, DERBY Spezialfutter GmbH), safflower oil (GEFRO Distelöl, GEFRO Reformversand Frommlet KG, Memmingen, Germany), and Lutalin (*cis*-9,*trans*-11, 10 g/d *trans*-10,*cis*-12 CLA, BASF SE; **EFACLA**, n = 8), from −9 wk to 9 wk relative to parturition. Samples of liver tissue were obtained via biopsy under local anesthesia on −3, 0, and 4 wk relative to parturition. An additional sample was obtained at slaughter (9 wk postpartum). Samples were immediately frozen in liquid nitrogen and stored at −80°C until RNA extraction.

Total RNA was extracted using Trizol reagent (catalog no. 15596026, Life Technologies) in combination with a PureLink RNA mini kit (catalog no. 12183018A, Invitrogen) following the manufacturer's instructions. The RNA quantity (1,579 ± 502 ng/µL) and purity (2.08 ± 0.02 of 260:280 ratio) were determined using a NanoDrop spectrophotometer (ND 1000, NanoDrop Technologies Inc., Wilmington, DE), whereas quality (8.8 ± 0.5 of RNA integrity number) was assessed using an Agilent 2100 Bioanalyzer (Agilent Technologies, Santa Clara, CA).

Complementary DNA synthesis was performed with 1 µg of RNA using a High-Capacity RNA-to-cDNA kit (catalog no. 4387406, Applied Biosystems) and 2 pg/µL of luciferase encoding reporter transcript (catalog no. L4561, Promega). Expression of *FFAR1, FFAR2*, *FFAR3*, *GPR84*, and *PPARD* were determined by real-time quantitative PCR (**RT-qPCR**) using the StepOne Real-Time PCR System (Applied Biosystems, Waltham, MA). All primers were previously validated and reported by different studies: *FFAR1*, *FFAR3*, and *GPR84* by Durand et al. (2024), *FFAR2* by Friedrichs et al. (2014), *FFAR4* by Agrawal et al. (2017), and *PPARD* by Goselink et al. (2013). Samples and controls were amplified in triplicate as follows: 10 min at 95°C, 40 cycles of 15 s at 95°C (denaturation), and 45 s at 62°C (*FFAR1*) or 60°C (other genes; annealing + extension). To account for technical variations, the mRNA abundance of each gene of interest was normalized using an exogenous spike-in mRNA (luciferase) as an internal reference gene (Bui et al., 2009; Bernard et al., 2025). The mRNA expression was calculated according to the formula: RT-qPCR Efficiency^−ΔCt^ (**Ct** = cycle threshold), where Efficiency = 10^(−1/slope)^, and ΔCt = Ct target gene –Ct luciferase. Genes were considered not expressed when average Ct ≥32.

Gene expression was analyzed using the MIXED procedure of SAS 9.4 (SAS Institute Inc., Cary, NC) with a model containing treatment, time, and their interactions as fixed effects, and cow within treatment as a random effect. The mRNA expression data were log_2_-transformed to comply with normal distribution of residuals. Repeated-measurement correlations were performed using the R package rmcorr (v0.7.0, Bakdash and Marusich, 2017). Statistical significance was declared at *P* ≤ 0.05 and tendencies at 0.05 < *P* ≤ 0.10.

A multilevel statistical analysis was used to take into account the longitudinal aspect of the study (Liquet et al., 2012). We performed a multilevel multiple factor analysis (**MFA**) to study the various links between variables that may influence FFAR liver expression and the metabolic adaptations of transition dairy cows supplemented or not with EFACLA (FactoMineR v2.11, Lê et al., 2008; factoextra v1.0.7, Kassambara and Mundt, 2020). Multiple factor analysis was carried out using 5 groups of variables, including energy parameters, blood metabolic indicators, liver gene expression, and liver proteomics, defined in the companion articles (Gnott et al., 2020; Vogel et al., 2020, 2021; Veshkini et al., 2022), plus the liver gene expression from the present study. A supplementary group with qualitative variables (treatment and time) was also included. Results for liver genes were kept separated because samples were processed in different laboratories, and although previous studies normalized mRNA expression using reference genes, the present study used luciferase. The MFA correlation circle showed the top 50 variables that contributed the most to the 2 first dimensions.

We observed expression of *FFAR1*, *FFAR2*, *FFAR3*, and *GPR84* in the liver of dairy cows from −3 to 9 wk relative to parturition (Figure 1), but *FFAR4* was not expressed. Previous studies have reported conflicting results regarding FFAR expression in the bovine liver. Friedrichs et al. (2014) observed expression of *FFAR1* and *FFAR2* but not *FFAR3* in multiparous and primiparous dairy cows. Agrawal et al. (2017) observed expression of *GPR84* but not *FFAR1* or *FFAR4* in multiparous dairy cows. Our findings are in agreement with a study in 9-mo-old beef bulls (Durand et al., 2024) and with RNA-sequencing in transition dairy cows (Gao et al., 2021). Overall, these studies indicate that FFAR are generally expressed at low levels in the liver of dairy cows. This low expression could help explain the conflicting results on liver expression, and their nondetection in untargeted proteomics analyses, as evidenced in our previous article (Veshkini et al., 2022).

**Figure 1:**
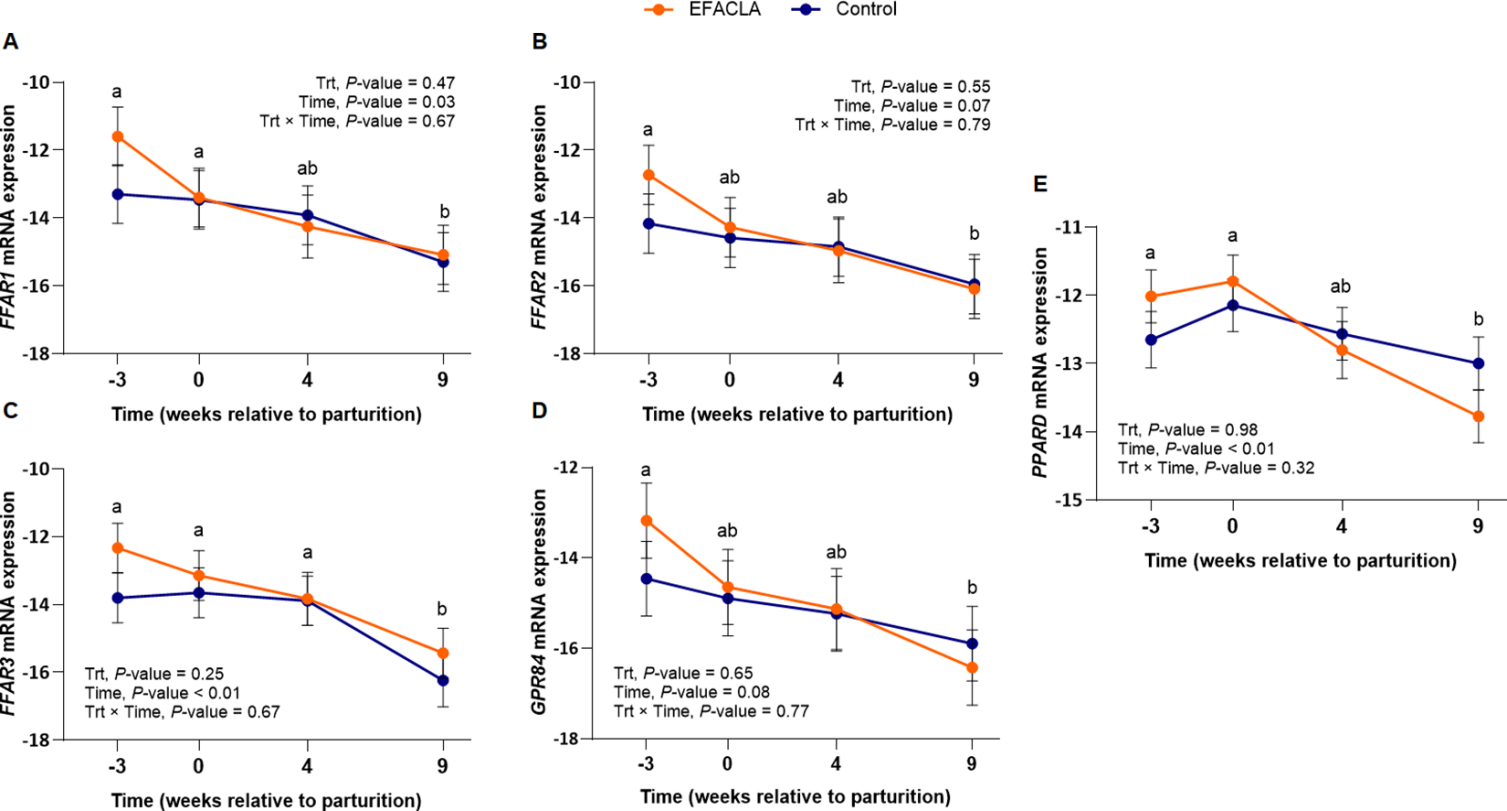
Liver expression of (A) *FFAR1*, (B) *FFAR2*, (C) *FFAR3*, (D) *GPR84*, and (E) *PPARD* in multiparous Holstein cows. Animals were abomasally infused with either coconut oil (control, n = 8) or EFA+CLA (EFACLA, n = 8), from −9 to 9 wk relative to parturition. The mRNA expression was calculated as RT-qPCR efficiency^−ΔCt^. Data are represented as log_2_-transformed (mean ± SEM). Different lowercase letters indicate significant differences between time points (*P* < 0.05).

*FFAR1* expression were greater (*P* ≤ 0.05) at prepartum (−3 wk) and parturition (0 wk) when compared with 9 wk postpartum (Figure 1A), and there was a tendency (*P* = 0.07) of greater expression at −3 wk than 4 wk relative to parturition. The mRNA levels of *FFAR2* and *GPR84* were greater (*P* ≤ 0.02) at −3 wk and tended (*P* = 0.07 and *P* = 0.08, respectively) to be greater at parturition when compared with 9 wk postpartum (Figure 1, Figure 1). *FFAR3* expression remained unchanged (*P* > 0.21) from −3 to 4 wk relative to parturition, then decreased (*P* = 0.02) at 9 wk postpartum (Figure 1C).

In the liver, Friedrichs et al. (2014) observed greater *FFAR1* at 3 wk prepartum than 3 wk postpartum in multiparous cows, with expression returning to prepartum levels by 15 wk into lactation, whereas *FFAR2* did not change over time. Decreased *FFAR4* expression was observed in the tailhead AT of dairy cows following parturition (Agrawal et al., 2017). Agrawal et al. (2017) suggested that this reduction might contribute to insulin resistance in the peripheral tissues, increasing glucose availability to the mammary gland. Lower FFAR expression postpartum found in our study could similarly have implications to metabolic adaptation, especially because insulin-sensitizing effects of FFAR are not restricted to the AT (Miyamoto et al., 2016). Different FFAR (e.g., FFAR1 and FFAR2) have been proposed to regulate hepatic insulin sensitivity in human and mouse models (Wu et al., 2012; Aoki et al., 2021). However, the mechanism underlying the reduced FFAR expression and their relation to insulin signaling remains unclear in bovines. In addition, Agrawal et al. (2017) indicated that decreased FFAR expression could serve as negative feedback to prevent receptor hyperactivation during periods of high ligand concentration. This is logical when considering the increases in plasma long and short-chain FA observed in dairy cows following parturition (Friedrichs et al., 2016; Vogel et al., 2020).

Current literature lacks information on the longitudinal *FFAR3* expression in dairy cows' liver. A hepatic transcriptome analysis showed no alterations in liver *FFAR3* when comparing −7 d and 16 d relative to parturition (Gao et al., 2021). In mice, FFAR3 signaling has been shown to be involved in starvation response by reducing sympathetic activity and energy expenditure (Rojas-Morales et al., 2016). These effects involve the inhibition of FFAR3 signaling by BHB (Kimura et al., 2011). In contrast, BHB was reported to activate FFAR3 in rats (Won et al., 2013). Thus, the metabolic effects of BHB through FFAR3 depend on whether BHB acts as agonist or antagonist, which has not yet been established for bovines. Future studies should investigate whether higher *FFAR3* expression around parturition contributes to the suppression of energy expenditure during negative energy balance of transition dairy cows.

We found no effects of EFACLA or its time interactions on FFAR liver expression (Figure 1). Friedrichs et al. (2014) supplemented CLA at a dose 2.5 times higher than the present study and found no effect of CLA on liver *FFAR1* or *FFAR2* in postpartum primiparous or multiparous cows. However, these authors observed greater *FFAR1* in the omental and retroperitoneal AT of primiparous cows at 105 DIM. Flaxseed oil, a source of α-linolenic acid, downregulated *FFAR1* in the liver but upregulated it in the skeletal muscle of 7-mo-old lambs (Duckett et al., 2019). As indicated in our previous article, EFACLA altered the overall plasma FA profile of supplemented cows, though few effects were observed in the FFA fraction (Gnott et al., 2020). α-Linolenic acid, which represents less than 1.2% of plasma FFA, was greater in EFACLA cows, whereas linoleic acid and CLA were unaffected. These results, together with very low lipoprotein lipase activity in the bovine liver which limits the release of FFA from lipoproteins (Hocquette et al., 1998), may help explain the lack of supplementation effect on FFAR liver expression observed in our study. Overall, these results highlight the complex regulation of FFAR transcription across tissues and the potential interplay of factors in this regulation, including the source and dosage of FA supplementation, along with animal species and age.

We observed strong positive correlations (r ≥ 0.70, *P* ≤ 0.01) in liver expression of FFAR, especially between *FFAR2* and *FFAR3* (r = 0.95), and between *FFAR2* and *GPR84* (r = 0.97). These strong correlations in mRNA expression may suggest synchronous regulation of FFAR at the transcriptional level, although the exact mechanism is still unknown. In addition, correlations between FFAR and EB, blood metabolic indicators, and other liver genes were generally weak, and few were significant. We observed a negative correlation between *FFAR2*, *FFAR3*, *GPR84*, and *PPARD* with the expression of insulin-like growth factor-binding protein 3 (*IGFBP3*; −0.30 ≥ r ≥ −0.32, *P* ≤ 0.05), and trends for negative correlation between of *FFAR1*, *FFAR2*, *FFAR3*, and *IGFI* expression (−0.26 ≥ r ≥ −0.29, *P* ≤ 0.10). *FFAR2* and *GPR84* were negatively correlated with plasma insulin-like growth factor-binding protein 2 (IGFBP-2; r = −0.31 and −0.37, respectively, *P* ≤ 0.03). Moreover, we found a negative correlation between *GPR84* and *PPARD* and energy intake (r = −0.33, *P* = 0.03) and fresh matter intake (**FMI**; r = −0.30, *P* = 0.05), respectively.

Liver *PPARD* was greater (*P* ≤ 0.03) at −3 wk and parturition than at 9 wk postpartum (Figure 1E), and it was moderately correlated with FFAR (0.41 ≥ r ≥0.51, *P* ≤ 0.01), with the strongest correlation being with *FFAR1*. In human HepG2 cells, the activation of PPARD through the FFAR1–phospholipase C–calcium pathway improved insulin sensitivity and increased β-oxidation in hepatocytes (Wu et al., 2012). Previous studies in dairy cows have shown that hepatic PPARD is activated and its expression increases with plasma NEFA (Busato and Bionaz, 2020). However, PPARD metabolic effects on the bovine liver remain unclear. In our study, we observed that *PPARD* expression peaked at parturition, when plasma NEFA were the highest (Vogel et al., 2020). Furthermore, we observed a moderate correlation between *PPARD* and *FFAR1*. Unlike in mice, however, we found no evidence linking *FFAR1* and *PPARD* with improved insulin sensitivity or β-oxidation.

The individual MFA plot (Figure 2A) showed good discrimination of time relative to parturition on the 2 first dimensions. This was expected when considering that FMI and DMI were the parameters that mostly contributed for dimension 1, whereas EB was the main contributor for dimension 2. The individual plot was put in relation to the variable plot (Figure 2B). At parturition, cows presented high levels of plasma NEFA and bilirubin, as well as high liver expression of *PPARD,* peroxisome proliferator-activated receptor gamma (*PPARG*), and FFAR*.* In contrast, these animals showed low levels of genes (e.g., *IGFI* and *INSR*) and plasma IGFBP-3, associated with insulin and somatotropic axis, along with genes related to gluconeogenesis (e.g., *G6PC*), ketogenesis (*HMGCS2*), FA β-oxidation (e.g., *CPT1*), and very low-density lipoprotein assembly (*MTTP*). Furthermore, we observed that increased plasma BHB at 4 wk postpartum was concomitant with a high plasma level of growth hormone (**GH**) and IGFBP-2. In contrast, at 4 wk postpartum, animals showed low plasma insulin, adiponectin, leptin, IGF-I, glucose, and triglycerides (Figure 2B).

**Figure 2:**
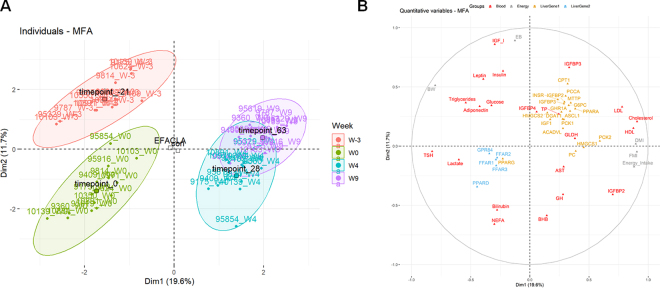
Multiple factor analysis (MFA). (A) Projection of individual samples according to time relative to parturition: −3 (red), 0 (green), 4 (blue), and 9 wk (purple). (B) Multiple factor analysis correlation circle, variable representation with energy zootechnical parameters (gray), blood metabolic indicators (red), liver genes defined in the companion articles (Gnott et al., 2020; Vogel et al., 2020, 2021; Veshkini et al., 2022; orange), plus liver genes from the present study (blue). Figure displays the top 50 quantitative variables contributing for the 2 first dimensions (Dim1 and Dim2, respectively). Due to the larger number of variables in the liver proteomics block, their individual contribution is low. However, some are strongly correlated with these 2 dimensions.

Our MFA results clearly illustrate the dynamics of metabolic and endocrinal adaptation around parturition, for example (1) the uncoupling of the somatotropic axis and the decreased insulin sensitivity are associated with increasing GH and IGFBP-2, and decreasing IGF-I, IGFBP-3, and insulin in the plasma (Chilliard, 1999); and (2) during early lactation, high-producing dairy cows exhibit lower plasma glucose, whereas NEFA and BHB concentrations increase (Grummer, 1995). We observed weak associations of liver FFAR with elements of the somatotropin axis. In fact, bovine and goat in vitro studies indicated that short-chain FA inhibit GH content and release in anterior pituitary cells (Ishiwata et al., 2000). This effect has been suggested to be exerted through FFAR2 and 3 (Wang et al., 2013). However, in our conditions, no obvious link between GH and *FFAR2* and *FFAR3* expression was observed. Overall, both multivariate (MFA) and univariate (correlation) analyses revealed weak links between liver FFAR and other metabolic parameters during the transition period.

Taken together, our study demonstrated the dynamics of *FFAR1*, *FFAR2*, *FFAR3*, and *GPR84* expression in the liver of multiparous dairy cows around parturition and early lactation. The decreased expression of FFAR in the liver from pre- to postpartum may reflect negative feedback that prevents receptors hyperactivation during periods of high FFA concentrations. Physiological relevance of this downregulation, as well as the individual contributions of FFAR to the hepatic metabolism, require further investigation.
